# Dr. Robert A. Wheaton

**Published:** 1898-04

**Authors:** 


### Obituary.

Dr. Robert A. Wheaton, of St. Paul, Minn., died February 13, 1898, aged 34 years. The *Western Medical Review* remarks upon this sad event as follows :

Dr. Wheaton had already become one of the most prominent surgeons of the west, and his death will be a great loss to the profession. His death was quite sudden. He had for years been suffering from some brain trouble, an abscess probably, and he was to have submitted to an operation two or three days previous to his death, but for some reason it was put off for a week. He had been out riding just before death came, and while it was known that his condition was serious, none looked for such an immediate sad ending. Dr. Wheaton had been married two years. He was a graduate of the St. Paul and Harvard medical colleges and had spent two years in the Massachusetts general hospital. He was clinical instructor in surgery at the University of Minnesota, a member of the American medical association, the Minnesota state medical society, Massachusetts state medical society, association of military surgeons of the national guard and of the local county society.

